# Taking sustainability of cardiac catheterization to the next level: the Hartplastic showcase

**DOI:** 10.1007/s12471-026-02045-7

**Published:** 2026-05-19

**Authors:** Steef J. Sinkeler, Mariska van Cronenberg, Sjoerd van Rietbergen, Pieter A. Vriesendorp, Renicus S. Hermanides, Migael Nieuwenhuis, Aukelien C. Dimitriu-Leen, Robin Nijveldt, Joost D. E. Haeck

**Affiliations:** 1 NHL Stenden University of Applied Sciences, Leeuwarden, The Netherlands; 2 Delft University of Technology, Delft, The Netherlands; 3 Maastricht University Medical Centre, Maastricht, The Netherlands; 4 Isala Hospital, Zwolle, The Netherlands; 5 Present Address: Frisius Medical Centre, location Leeuwarden, Leeuwarden, The Netherlands; 6 Radboud Medical University Centre, Nijmegen, The Netherlands

**Keywords:** Sustainability, Cardiac procedures, Cardiac catheterization, Medical waste, Recycling

## Abstract

**Background:**

Reducing the environmental impact of healthcare is paramount to achieving zero-emission healthcare services. Considering the increasing amount of waste that is produced by cardiac procedures, we aimed to investigate whether plastic waste from these procedures could be recycled and repurposed into new medical instruments. To facilitate this process, the HartPlastic Foundation was established for research and awareness purposes.

**Methods:**

Primarily, the non-contaminated materials found in medical packaging were separated, identified, and rated based on suitability for recycling. Glycolized polyethylene terephthalate (PET-G) was found to be the most promising material, which comprised roughly 10% of plastic waste.

**Results:**

In a process that involved cleaning, shredding, drying, spooling, and 3D-printing, PET‑G was then repurposed into different products, such as bed hooks that could be used in the catheterisation laboratory, and a flashlight that could be used by nurses on night shifts.

**Conclusion:**

These results could help reduce the environmental impact of procedures in the catheterisation laboratory by providing a locally scalable recycling process for medical waste to help reach the goal of zero healthcare-related carbon emissions.

## What’s new?

- First paper to show that medical waste, specifically PET‑G, from the cath lab can be reduced by recycling locally to produce new medical products for the cath lab.
- Recycling locally prevents recycled plastic from amassing in storage facilities.
- Scalable process that can be applied to any hospital.

## Introduction

The healthcare sector globally accounts for approximately 4% to 5% of worldwide greenhouse gas emissions, and in the Netherlands, this number is even higher at 7% [1]. The exponential growth of medical waste poses significant challenges to environmental sustainability, with a projected threefold increase in mass by 2050 [2]. When looking at cardiology specifically, procedures in the cardiac catheterisation laboratory (CCL) generate a considerable amount of waste during each procedure. Most of this waste is derived from packaging material for catheters, stents, devices, and other equipment that waste needs to adhere to strict regulations regarding sterility.

The European Green Deal stated that all packaging in the European Union should be recyclable or reusable by 2030 [3]. Nevertheless, insufficient attention has been paid to quantifying and reducing waste from CCLs to help lower the carbon footprint of this packaging material and decrease the amount of plastics released into nature [4].

While CCLs have established a routine waste management protocol in daily practice, this protocol is not typically employed to separate recyclable waste from non-recyclable waste. In most CCLs, all contaminated waste is collected in designated bags, shredded, and subsequently placed in disposal bags that are incinerated. Non-contaminated waste, including paper, plastics, and cardboard boxes (packaging from balloon catheters, stents, guides, etc.), is collected in ‘coloured’ bags. Much of this potentially recyclable waste ends up in landfills due to the absence of a formal recycling programme. Evaluating invasive cardiac procedures, Doshi et al. [2] quantified the amount of recyclable waste and observed approximately 1.4 kg of recyclable waste with a percutaneous coronary intervention (PCI) and about 0.7 kg of recyclable waste with a diagnostic right heart catheterization (RHC). A study by a team at Stanford noted that 15% of waste per procedure is recyclable, which could translate to 12 tons of material diverted from landfills annually just from one CCL [5]. Globally, this could add up to 150 million tonnes of waste from cardiovascular procedures, of which 25% could be recyclable [6].

To support our ambitions of moving towards zero-emission healthcare services, we founded the Hartplastic Foundation to establish a framework for implementing a recycling process. To achieve sustainability at the CCL without the necessity for complex logistics, this study explored the possibilities for recycling, or rather upcycling, non-contaminated medical waste into medical products that could be reintroduced into healthcare directly.

## Methods

The process of exploring recycling possibilities involved various steps, including:

- Identifying the most promising opportunities for recycling or upcycling materials.
- Studying the waste collection process to separate the most promising materials
- Identifying production techniques for transforming these materials into products
- Exploring the most promising medical product options by producibility and scalability
- Prototyping medical products and their potential production processes

Consequently, as one of the initial practical steps, we conducted an analysis of the uncontaminated plastics from the procedures at the CCL and examined whether these materials are suitable for recycling, and if so, whether it would be feasible to utilise the recyclable raw materials for new sustainable products intended for use in our hospitals.

Firstly, for analysing all the plastics used during catheterisation procedures, waste was collected from four CCLs (Frisius Medical Center, Leeuwarden, the Netherlands; Isala Hospital, Zwolle, the Netherlands; Radboud University Medical Center, Nijmegen, the Netherlands; and Maastricht University Medical Center, Maastricht, the Netherlands) to quantify the amount of uncontaminated recyclable plastic material utilised. Procedures included coronary angiogram (CAG), percutaneous coronary intervention (PCI), diagnostic right heart catheterisation (RHC), and electrophysiological studies (EPS).

Throughout a workday, non-contaminated recyclable materials (plastics) were gathered during all cardiac catheterisation procedures. The materials were weighed in kilograms (kg) and labelled by categories of plastics. Materials incorrectly identified as recyclable or contaminated by blood or bodily fluids were removed before weighing.

The estimates of plastic fractions were conducted using both labelling on the plastic, if available, and polymer densitometry by the National Test Centre for Circular Plastics (NTCP), an independent centre for innovation and research in the Netherlands. Data were processed using Excel 16 (Microsoft Corporation, Redmond, WA, USA).

## Recycling process

Among all these non-contaminated plastics, glycolized polyethylene terephthalate (PET-G) can already be commonly (mechanically or chemically) recycled. Consequently, PET‑G was selected for recycling into a new raw material that can be employed for various new products (ranging from low to high-end) intended for use in hospitals. With financial backing from Samenwerkingsverband Noord-Nederland (SNN) and the city council of Leeuwarden, alongside the Department of Circular Plastics at the local university in Leeuwarden (NHL Stenden, Leeuwarden & Emmen) and the Department of Industrial Design at the Technical University of Delft (TU Delft), research was conducted on the recycling process and potential applications for the recycled material, aiming to close the circular loop of PET‑G (non-contaminated) plastic waste.

**Fig. 1:**
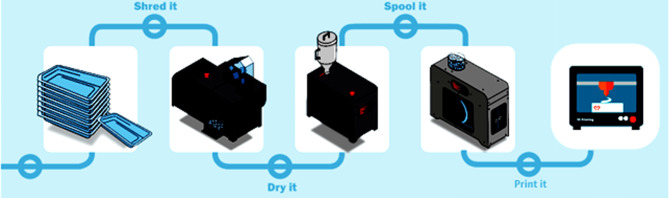
Recycle process of non-contaminated PET plastics from CCL

To summarise this recycling process, firstly, the PET‑G plastic waste was separated directly at the hospital from other plastics in a separate bin. The plastic was disposed of directly after removing the necessary tooling from the packaging and was transported to the NHL research facility for cleaning and cutting away glue residue by hand before mechanical shredding by a plastic shredder (GP20 Plastic Shredder Hybrid, 3devo, Utrecht, the Netherlands), turning it into flakes. Thereafter, these plastic PET‑G flakes are dried for four hours at a temperature of 60 degrees Celsius to remove any moisture using a special dryer for plastics (Airid polymer dryer, 3devo, Utrecht, the Netherlands). Finally, once dried, the flakes are manually deposited into the filament maker (3devo Precision 350, 3devo, Utrecht, The Netherlands) to produce filament suitable for use in a 3D printer (UltiMaker S3, UltiMaker, Geldermalsen, The Netherlands). This recycling process is illustrated in Fig. 1.

**Fig. 2:**
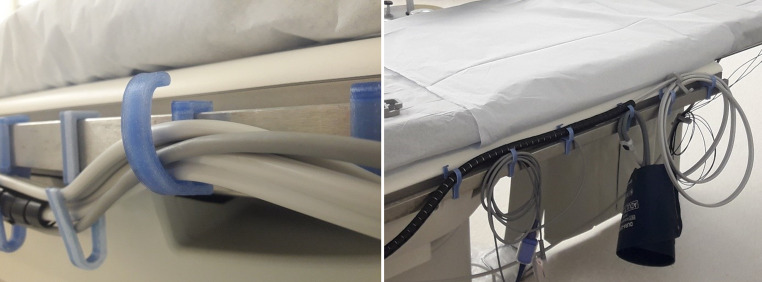
Several redesigned plastic waste bed hooks for cables used at the operating table within the CCL

**Fig. 3:**
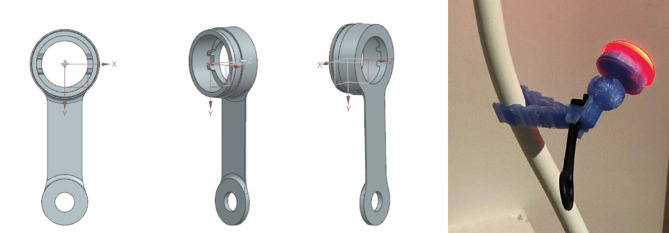
Prototype design and finalized 3D-printed prototype of the flashlight for nurses (suitable for both 3D printing and injection moulding)

After several competitions (via the thesis of the student minor in Industrial Design, TU Delft) and contemplations, low- and high-end prototype products were designed and fabricated. Firstly, a low-end product, a redesigned bed hook for cables at the operating table in the CCL, was made and tested (Fig. 2). Moreover, a prototype for a dedicated flashlight for nurses in the hospital was 3D-printed and could be suitable for upscale production (Fig. 3). The final prototype of the flashlight was distributed among nurses on the cardiology ward for use in their daily practice to obtain real-world feedback on its strengths and weaknesses.

To be able to make this process more scalable and create more advanced high-end products in the future, additional collaboration with the TU Delft was sought. This led to refinement of the 3D-printing process, amongst others, by utilizing a temperature tower and a retraction tower. These results helped calibrate the equipment more precisely, allowing for an optimized 3D-printing process and a more sturdy end product.

## Results

### Analysis of non-contaminated plastics

On a randomly chosen day, that was deemed to be representative of a typical working day, in each participating center (Frisius Medical Center, Isala Hospital, Maastricht University Medical Center, and Radboud University Medical Center), all non-contaminated plastics were collected from all operating CCLs (range 4–5 CCLs per center) in separate bags during the complete working day. Procedures performed included coronary angiograms (CAG), percutaneous coronary interventions (PCI), and electrophysiological studies (EPS). Chronic Total Occlusion (CTO) procedures, Transcatheter Aortic Valve Implantations (TAVI), and other transcatheter valve procedures, as well as cardiac device implantations were not included. The number of procedures per center was not recorded.

A median of 4.5 kilograms (kg) (range 4.5–6.0 kg) of non-contaminated plastics was collected per center on a working day for all 4 or 5 CCLs combined, amounting to 2–3 bags of non-contaminated plastic. The amount of contaminated plastics was not measured.

In the Netherlands, there are approximately 49 operational catheterisation centers that follow similar procedures. If these findings are extrapolated to all catheterisation laboratories in the Netherlands, assuming 250 regular working days, the non-contaminated plastic waste stream from regular procedures amounts to about 30,625 bags per year, equating to 55 tonnes of non-contaminated, recyclable plastic annually. Extrapolating the results to include transcatheter valve procedures, device implantations, and emergency procedures outside regular working hours and days, which would roughly double the amount of plastics, this would equate to 61,000 bags of plastic, or 110 tonnes of non-contaminated plastic annually.

The collected non-contaminated plastic waste stream was analysed to identify the various plastic polymers present (Tab. 1). In terms of mass, the majority of the plastics consisted of low-density polyethylene (LDPE) foils, comprising approximately 46%. Significant fractions included high-density polyethylene (HDPE), glycolyzed polyethylene terephthalate (PET-G), and polystyrene (PS), making up 13%, 10%, and 8% (or 18.9 tonnes, 14.3 tonnes, and 11.5 tonnes annually), respectively. To facilitate the separation of the different plastics, the types contained in each packaging material were identified.

**Table 1:** Analysis of the several fractions of non-contaminated waste as performed at the National Test Centre Circular Plastics.

	Percentage non-contaminated plastic waste (median [range] %)	Tonnes of material per year extrapolated to all catheterisation laboratories in the Netherlands (*1000 kg)
HDPE	13 [10–15]%	18.9
*PET*	* 10 [10–15]%*	*14.3*
Tyvek	15 [10–15]%	21.6
PE foil (LDPE)	46 [45–50]%	66.7
PC	< 1 [0–5]%	0.1
ABS	< 1 [0–1]%	0.4
PS	8 [8–10]%	11.5
PP	3 [1–5]%	4.7
PVC/C	< 1 [0–1]%	1.3
Alu multilayer	< 1 [0–1]%	1.4
Rest undefined	2 [2–5]%	2.9
Paper	1 [1–2]%	2.1

*HDPE* high-density polyethylene, *PET* polyethylene terephthalate, *LDPE* low-density polyethylene, *PC* polycarbonate, *ABS* acrylonitrile butadiene styrene, *PS* polystyrene, *PP* polypropene, *PVC/C* polyvinylchloride

### Testing of printed products

Three prototypes of the flashlight were distributed among the nursing staff of the cardiology ward at Frisius Medical Center in Leeuwarden, and questionnaires for feedback were distributed.

Positive findings were that the flashlight was practical and useful in daily practice, especially the fact that it was attachable to the breast pocket of the nursing uniform.

Negative findings were that the light itself was not powerful enough and lacked a dimming function, that the light could not rotate further, and that the design of the clip for attaching to the uniform was deemed too flimsy, although no failure was reported.

## Discussion

This is, to our knowledge, the first study to explore the recycling of plastic waste in a CCL and its use for 3D printing. Based on our results, roughly 150 tonnes of non-contaminated plastics are deposited in landfills annually. PET‑G packaging was chosen as the most suitable plastic for recycling, as it offers good mechanical recycling properties and is compatible with 3D printing and injection moulding for circular applications. Just by recycling PET‑G packaging, using the methods outlined in this study, an estimated 10%, or 15 tonnes of plastics, could be recycled and repurposed in the Netherlands alone. Considering that using recycled PETG may reduce the CO2 footprint of 3D-printing by 2.19 [7] to 3.30 [8] kg CO2/kg, repurposing these 15 tonnes of plastic may reduce the CO2 footprint of all CCLs in the Netherlands by approximately 32.9 to 45.9 tonnes of CO2.

A study by Amin et al. [6] estimated that 150,000,000 kg of waste are generated from cardiac procedures every year, with 25% of that waste being potentially recyclable. Based on these numbers, assuming that PET‑G makes up 10% of all recyclable waste, this would amount to saving 3.75 million kg from the landfill and saving 8.2125 to 12,375 tonnes of CO2 from being released in the atmosphere.

Our initiative demonstrates the potential to recycle non-contaminated PET‑G plastics generated from the CCL into new sustainable products for use in hospitals. However, further research and innovation towards the implementation of a ‘circular economy’ of waste to prevent, minimise, or completely reuse waste in the healthcare context is necessary to move away from the traditional plastic management model (production-use-disposal) that is common practice. With millions of procedures performed worldwide (with an estimated 5 million CCL (including 250,000 TAVI) procedures worldwide) [6], a commitment to implement even small changes in daily practice by the cardiology community could lead to significant environmental benefits. Waste management, including incineration or autoclaving of contaminated waste, and healthcare solid waste that ends up in landfills, accounts for 3% of (or 40% of the total) greenhouse gas emissions [1]. Particularly, with increasing amounts of waste produced by an ever-growing number of ever-more complex invasive cardiac procedures, this quantity of waste is unlikely to diminish in the near future.

While our process is by no means the perfect recycling solution—since this would require a closed loop in which the recycled PET‑G is used to produce new packaging for CCL equipment—it demonstrates that at least repurposing plastics is possible, even in a small setting with limited resources. Additionally, it emphasises that even in a complex environment, such as a hospital, recycling is feasible, despite not being an integral part of the initial primary processes.

The findings of our studies could help other hospitals investigate those delineated options, such as identifying packaging materials suitable for recycling, identifying products made from virgin plastics that could be produced in-house instead of being purchased, producing filament for 3D printing in a nearby facility, or designing new medical products for use in healthcare. To help other hospitals set up their own closed-loop system, the technical aspects have been detailed in another article [9].

To facilitate a more efficient production process, a logical next step would be to study the options for injection moulding of PET-G/PET and various other plastics, such as HDPE and LDPE. This could help achieve a bigger scale and increase the impact of recycling on the reduction of carbon emissions.

However, these processes have their own pitfalls and obstacles, which we are dedicated to overcoming. This could result in further improvements that could contribute to our planet-sustainability mission. Nevertheless, scaling up these recycling activities nationwide would involve even bigger challenges, such as production facilities, durable product design, transportation, and distribution, which would impact our present overall healthcare processes.

Several countries, including the Netherlands, have taken steps to reduce healthcare emissions in line with the Paris Agreement’s goal of achieving net-zero emissions by 2050. The guiding principle of ‘reduce, reuse, and recycle’ is relevant for implementation in the catheterisation laboratory [9].

1. Reduce wastage by removing non-essential items from procedural kits and avoiding opening items that will not be used. Also, avoid inventory mismanagement (e.g., losing expired items), and wasteful use of water and paper.
2. Reuse items that are safe to desterilise after appropriate infection control clearance. Sterile single-use items like catheters, balloons, manifolds, and syringes cannot be reused, but items like plastic bowls can.
3. Recycle 100% of the uncontaminated plastics, paper, and cardboard boxes from cardiac procedures. Segregating recyclable waste and using the right waste management practices diverts materials away from landfills and incineration.

Implementing this practice is an easy step towards achieving a sustainable Heart Center through her services. Even the ultimate goal, such as completing the PET‑G recycling loop as outlined in this article, is attainable, especially given the urgent need to focus on decarbonising the healthcare sector. However, this necessitates the packaging sector to find methods for collecting discarded packaging before it reaches landfills and incineration plants. The study project highlights the importance of developing and implementing cost-effective and safe recycling methods to reduce the carbon footprint and promote environmental sustainability in healthcare. Establishing ‘green teams’ that conduct waste audits and identify ways to apply the principle of ‘reduce, reuse, and recycle’ could serve as an initial step to assist our planet [6].

## Limitations

Our analysis and recycling process has several limitations. Firstly, our plastic waste analyses were conducted on random workdays at the catheterisation laboratories, irrespective of the procedures planned for that day. Thus, the extrapolation of these numbers may lead to compounding errors in calculating the amount of non-contaminated, recyclable plastic. Secondly, PET‑G plastic waste constitutes a relatively small fraction of the total waste generated, and thus its impact will be minimal, although our calculations show that it could still lead to significant reductions in carbon emissions on a global level. However, PET‑G packaging material showed its potential for recycling and 3D printing. Thirdly, separating waste is not the core business of any CCL, and as a result, contaminated waste could easily compromise the non-contaminated fraction if not separated properly.

More studies are needed to quantify the volume of (non-)contaminated waste in different settings, hospitals, and countries. A limitation of our study is therefore the lack of an objective cost analysis or, indeed, a formal Life Cycle Analysis to be able to extrapolate the impact of our results if implemented on a larger scale. These analyses are also needed to quantify the environmental impact of recycling and its financial effects (i.e., cost savings). While this has been attempted in a similar study [10], our data did not allow for a proper cost-benefit analysis. Finally, scaling up a cost-effective recycling strategy for non-contaminated waste has not been organized in most Western countries. Implementing a successful recycling strategy requires not just cost-effectiveness, but also time and dedication from CCL personnel, who more often than not, will not be compensated for their efforts, as well as an organization dedicated to reducing its carbon footprint.

And even then, successful recycling is not a success by itself. In fact, the market for recycled plastics is not growing due to a lack of competitiveness with newly produced (virgin) plastics. Thus, repurposing these recycled plastics is just as critical as recycling in itself.

## Conclusions

As healthcare professionals, we still have ‘Big, hairy, audacious goals’ to decarbonize our health system and move towards zero-emission services. Our initiative shows the possibilities for the first steps in closing the plastic waste loop at a catheterization laboratory. We hope that our achievements will encourage our peers to reflect on their own practice and commit to becoming ‘greener’ professionals.

## Data Availability

Data are available on request from the corresponding author.
